# Validation of administrative health data for the identification of endometriosis diagnosis

**DOI:** 10.1093/humrep/deae281

**Published:** 2024-12-20

**Authors:** A C Kiser, R Hemmert, R Myrer, B T Bucher, K Eilbeck, M Varner, J B Stanford, C M Peterson, A Z Pollack, L V Farland, K C Schliep

**Affiliations:** Department of Biomedical Informatics, University of Utah, Salt Lake City, UT, USA; Department of Family and Preventative Medicine, University of Utah, Salt Lake City, UT, USA; Department of Family and Preventative Medicine, University of Utah, Salt Lake City, UT, USA; Department of Biomedical Informatics, University of Utah, Salt Lake City, UT, USA; Division of Pediatric Surgery, Department of Surgery, University of Utah School of Medicine, Salt Lake City, UT, USA; Department of Biomedical Informatics, University of Utah, Salt Lake City, UT, USA; Division of Reproductive Endocrinology and Infertility, Department of Obstetrics and Gynecology, University of Utah, Salt Lake City, UT, USA; Department of Family and Preventative Medicine, University of Utah, Salt Lake City, UT, USA; Division of Reproductive Endocrinology and Infertility, Department of Obstetrics and Gynecology, University of Utah, Salt Lake City, UT, USA; Department of Global and Community Health, College of Public Health, George Mason University, Fairfax, VA, USA; Department of Epidemiology and Biostatistics, Mel and Enid Zuckerman College of Public Health, University of Arizona, Tucson, AZ, USA; Department of Obstetrics and Gynecology, College of Medicine—Tucson, University of Arizona, Tucson, AZ, USA; Department of Family and Preventative Medicine, University of Utah, Salt Lake City, UT, USA

**Keywords:** endometriosis, administrative health data, electronic health records, validation, endometriomas, diagnosis codes

## Abstract

**STUDY QUESTION:**

How do endometriosis diagnoses and subtypes reported in administrative health data compare with surgically confirmed disease?

**SUMMARY ANSWER:**

For endometriosis diagnosis, we observed substantial agreement and high sensitivity and specificity between administrative health data—International Classification of Diseases (ICD) 9 codes—and surgically confirmed diagnoses among participants who underwent gynecologic laparoscopy or laparotomy.

**WHAT IS KNOWN ALREADY:**

Several studies have assessed the validity of self-reported endometriosis in comparison to medical record reporting, finding strong confirmation. We previously reported high inter- and intra-surgeon agreement for endometriosis diagnosis in the Endometriosis, Natural History, Diagnosis, and Outcomes (ENDO) Study.

**STUDY DESIGN, SIZE, DURATION:**

In this validation study, participants (n = 412) of the Utah operative cohort of the ENDO Study (2007–2009) were linked to medical records from the Utah Population Database (UPDB) to compare endometriosis diagnoses from each source. The UPDB is a unique database containing linked data on over 11 million individuals, including statewide ambulatory and inpatient records, state vital records, and University of Utah Health and Intermountain Healthcare electronic healthcare records, capturing most Utah residents.

**PARTICIPANTS/MATERIALS, SETTING, METHODS:**

The ENDO operative cohort consisted of individuals aged 18–44 years with no prior endometriosis diagnosis who underwent gynecologic laparoscopy or laparotomy for a variety of surgical indications. In total, 173 women were diagnosed with endometriosis based on surgical visualization of disease, 35% with superficial endometriosis, 9% with ovarian endometriomas, and 14% with deep infiltrating endometriosis. Contemporary administrative health data from the UPDB included ICD diagnostic codes from Utah Department of Health in-patient and ambulatory surgery records and University of Utah and Intermountain Health electronic health records.

**MAIN RESULTS AND THE ROLE OF CHANCE:**

For endometriosis diagnosis, we found relatively high sensitivity (0.88) and specificity (0.87) and substantial agreement (Kappa [*Κ*] = 0.74). We found similarly high sensitivity, specificity, and agreement for superficial endometriosis (n = 143, 0.86, 0.83, *Κ * = 0.65) and ovarian endometriomas (n = 38, 0.82, 0.92, *Κ * = 0.58). However, deep infiltrating endometriosis (n = 58) had lower sensitivity (0.12) and agreement (*Κ * = 0.17), with high specificity (0.99).

**LIMITATIONS, REASONS FOR CAUTION:**

Medication prescription data and unstructured data, such as clinical notes, were not included in the UPDB data used for this study. These additional data types could aid in detection of endometriosis. Most participants were white or Asian with Hispanic ethnicity reported 11% of the time, which may limit generalizability to some US states. Additionally, given that participants whose administrative health records we utilized were also part of the ENDO Study, the surgeons may have been more vigilant in diagnostic coding due to the operative forms they completed for the ENDO Study, which may have led to increased validity. However, the codes compared in the UPDB would have been entered by medical coders as part of standard clinical practice.

**WIDER IMPLICATIONS OF THE FINDINGS:**

We observed substantial agreement between administrative health data and surgically confirmed endometriosis diagnoses overall, and for superficial and ovarian endometrioma subtypes. These findings may provide reassurance to researchers using administrative healthcare records to assess risk factors and long-term health outcomes of endometriosis. Our findings corroborate prior research that demonstrates high specificity but low sensitivity for deep infiltrating endometriosis, indicating deep infiltrating endometriosis is not reliably annotated in administrative healthcare data. This suggests that medical record-based deep infiltrating endometriosis diagnoses may be suitable for etiologic studies but not for surveillance or detection studies.

**STUDY FUNDING/COMPETING INTEREST(S):**

The original ENDO Study was funded by the Intramural Research Program, Eunice Kennedy Shriver National Institute of Child Health and Human Development, National Institutes of Health (contracts NO1-DK-6-3428; NO1-DK-6-3427; 10001406-02). We acknowledge partial support for the UPDB through grant P30 CA2014 from the National Cancer Institute, University of Utah and from the University of Utah’s program in Personalized Health and Center for Clinical and Translational Science. This research was also supported by the NCRR grant, ‘Sharing Statewide Health Data for Genetic Research’ (R01 RR021746, G. Mineau, PI) with additional support from the Utah Department of Health and Human Services, University of Utah. Additionally, this research was supported by the Utah Cancer Registry, which is funded by the National Cancer Institute’s SEER Program, Contract No. HHSN261201800016I, the US Centers for Disease Control and Prevention’s National Program of Cancer Registries, Cooperative Agreement No. NU58DP007131, with additional support from the University of Utah and Huntsman Cancer Foundation. Research reported in this publication was also supported by the National Institutes of Health (Award Numbers R01HL164715 [to L.V.F., K.C.S., and A.Z.P.] and K01AG058781 [to K.C.S.]), by the Huntsman Cancer Institute’s Breast and Gynecologic Cancers Center, and by the Doris Duke Foundation’s COVID-19 Fund to Retain Clinical Scientists funded by the American Heart Association. A.C.K. was supported by Training Grant Number 5T15LM007124 from the National Library of Medicine to K.E. The content is solely the responsibility of the authors and does not necessarily represent the official views of the National Institutes of Health or other sponsors. There are no competing interests among any of the authors.

**TRIAL REGISTRATION NUMBER:**

N/A.

## Introduction

Endometriosis is a common gynecological disorder affecting at least 11% of reproductive-age women in community-based samples (Buck Louis *et al.*, 2011) but increasing to approximately half of women experiencing pelvic pain (Schliep *et al.*, 2015) or infertility (Buck Louis *et al.*, 2016), the two most common comorbidities associated with endometriosis. Emerging research suggests that a diagnosis of endometriosis may be associated with a higher likelihood of a subsequent diagnosis of ovarian cancer (Wang *et al.*, 2016), autoimmune disease (Shigesi *et al.*, 2019), or cardiovascular disease (Mu *et al.*, 2017). The time lag between women’s early reproductive health and mid-life chronic disease risk, in addition to the limited size of many reproductive epidemiologic studies, makes routine electronic health record (EHR) data and administrative health data a promising vehicle for carrying out risk factor research. However, before such research can be undertaken, an understanding of how well administrative health data accurately capture endometriosis within population-based cohorts is needed (Benchimol *et al.*, 2016).

Prior research has focused on assessing the reliability between and within gynecologic surgeons for diagnosing endometriosis (Schliep *et al.*, 2012, 2017) and the validity of self-reported endometriosis compared to administrative health records (including medical records and inpatient hospital registries) (Shafrir *et al.*, 2021). Indeed, our own prior research within the Endometriosis, Natural History, Diagnosis and Outcomes (ENDO) Study found substantial inter- and intra-rater reliability of endometriosis diagnoses between surgeons at different levels of expertise (Schliep *et al.*, 2012) in addition to increased inter-rater agreement when operative reports and/or pathology reports accompany operative images (Schliep *et al.*, 2017). Regarding validity, findings to date show a wide range of agreement between self-report of endometriosis and medical records from 32% to 89% (Missmer *et al.*, 2004; Saha *et al.*, 2015, 2017; Shafrir *et al.*, 2021). The variability in validation proportions could partially be due to varying populations, questionnaires, and data sources. Recent research evaluated four international cohorts’ self-reported endometriosis (n = 827 total) against clinically and surgically confirmed endometriosis and observed concordance of 84% and ≥95%, respectively (Shafrir *et al.*, 2021).

There is a research gap in comparing surgically confirmed endometriosis diagnoses to diagnoses recorded in administrative health data within nested cohorts. Therefore, we set out to compare diagnoses (test method) of endometriosis and endometriosis typology (superficial endometriosis [SE], ovarian endometriomas [OE], and deep infiltrating endometriosis [DE]) recorded in administrative health data with surgically confirmed diagnoses (reference method) in the same individuals who took part in the US-based ENDO Study (Buck Louis *et al.*, 2011).

## Materials and methods

### Study population

This study sample comprised participants from the ENDO Study (2007–2009) who were part of the Utah operative cohort (n = 412) and who could be linked to the Utah Population Database (UPDB) (100% linkage) (Buck Louis *et al.*, 2011). The ENDO Study included adult women undergoing laparoscopy or laparotomy for a variety of surgical indications, including pelvic pain (44%), pelvic mass (16%), irregular menses (13%), fibroids (10%), tubal ligation (10%), and infertility (7%). Surgeries were scheduled independently of and prior to ENDO Study enrollment. Women were excluded from the original ENDO Study if they were previously surgically diagnosed with endometriosis, pregnant, currently breastfeeding for 6 months or more, given injectable hormones within the past 2 years, or diagnosed with cancer other than non-melanoma skin cancer.

### Endometriosis diagnoses from surgically confirmed disease (reference method)

We used the established clinical gold standard definition for endometriosis, namely, surgically visualized disease (American Society for Reproductive Medicine, 1997; Kennedy *et al.*, 2005). All participating surgeons of the ENDO Study had surgical training in the diagnosis and staging of endometriosis (Buck Louis *et al.*, 2011; Schliep *et al.*, 2012). Consistent with the observational design, surgeons were not asked to change their practice in any way but were encouraged to obtain specimens for histology. Surgeons completed the revised American Society for Reproductive Medicine (rASRM) standardized operative report immediately after surgery to capture gynecologic and pelvic pathology including endometriosis, uterine fibroids, pelvic adhesions, benign ovarian cysts, neoplasms, and congenital Mullerian anomalies.

Endometriosis typology was assessed via the rASRM standardized form (American Society for Reproductive Medicine, 1997). Out of the 173 women with an endometriosis diagnosis, 168 (97%) women had information on lesion location and size recorded on the rASRM form. Women with only superficial lesions on the ovary or peritoneum were considered to have SE, deep lesions (>5 mm invasion) (Johnson *et al.*, 2017) noted in the peritoneum or obliteration of posterior cul-de-sac were considered to be DE, and deep lesions of any size noted in the ovary were considered to be OE; women who had deep ovarian and peritoneal lesions were considered to have OE + DE. Women without information on lesion location, size, and depth (n = 10) were assumed to have SE, as documented in prior research (Byun *et al.*, 2020).

### Endometriosis diagnoses from administrative health data (test method)

All participants in this study were diagnosed with endometriosis via laparoscopy or laparotomy as part of the previous ENDO Study (Buck Louis *et al.*, 2011). These diagnoses became part of the patient’s EHR via the normal administrative process of diagnosis code input and billing. We linked ENDO Study participants to the UPDB (Smith *et al.*, 2022). The UPDB is a unique database containing linked data on over 11 million individuals, including statewide ambulatory and inpatient records, state vital records, and University of Utah (UU) Health and Intermountain Healthcare (IH) EHRs—including diagnosis and billing codes—capturing the majority of Utah residents. UPDB endometriosis diagnoses were identified via International Classification of Diseases (ICD) 9 and 10 codes (617.X or N80.X). Our categorization was informed by previously published research (Saavalainen *et al.*, 2018; Rossi *et al.*, 2021) and expert opinion from our physician advisors (C.M.P. and J.B.S.) to determine the subtype of endometriosis based on the ICD code (see Supplementary Table S1). Data from the UPDB were restricted to the ENDO Study period (2007–2009). Medical record diagnoses that occurred more than 1 month after the individual’s participation in the ENDO Study were removed. We determined this 1-month threshold with expert opinion (consensus from obstetrics and gynecology providers) and timeline visualization (Supplementary Fig. S1).

### Diversity analysis

We isolated specific patient populations in our data to evaluate if disparities in validity exist between different groups. We compared endometriosis diagnoses of Women of Color to White women and women of Hispanic ethnicity to women of Non-Hispanic ethnicity.

### Statistical analysis

We compared patient characteristics between the women with and without endometriosis as per the ENDO Study research diagnoses. We used several metrics to measure the accuracy of the administrative health data diagnoses, as compared to surgically confirmed diagnoses, including percent agreement, area under the receiver operating characteristic curve (AUCR), sensitivity, specificity, negative predictive value (NPV), positive predictive value (PPV), and Cohen’s kappa (Κ). Additionally, we compared the endometriosis subtypes diagnosed from each source and calculated the accuracy measures. To calculate 95% confidence intervals, we bootstrapped the data 1000 times, building a distribution of measures. We calculated the confidence intervals from this distribution using the percentile method (Lee and Fung, 1993).

### Error analysis

We completed an error analysis to identify the patients with incorrect endometriosis diagnosis in the test method. For false negatives (those with surgically confirmed disease not reported in the administrative health data), we identified the diagnosis codes present in the administrative health data at the time of the study—when we expected the endometriosis diagnosis to be present. For false positives (those without disease per surgical confirmation but a record of disease in the administrative health data), we identified the endometriosis subtype present in the administrative health data at the time of the study—when we did not expect an endometriosis diagnosis.

## Results

### Patient characteristics as reported in the ENDO study

Table 1 describes the patient’s characteristics. Women with endometriosis had lower BMI (26.5 kg/m^2^) compared to women without endometriosis (29.5 kg/m^2^). Women with endometriosis were more likely to be nulliparous (52.0%) compared to women without endometriosis (29.7%). No other appreciable differences between diagnoses were identified. Stage I was the most common severity of endometriosis (56.6%) and SE was the most common subtype (82.7%).

**Table 1.** Demographics of the Utah site Endometriosis, Natural History, Diagnosis, and Outcomes Study (ENDO) operative cohort (n = 412).

Patient characteristics	Total population	Endometriosis (n = 173, 42.0%)	No endometriosis (n = 239, 58%)
Age, mean (SD)	32.6 (7.0)	31.6 (6.7)	33.3 (7.2)
BMI, mean (SD)	28.2 (8.2)	26.5 (7.4)	29.5 (8.6)
Parity			
Nulliparous, n (%)	161 (39.1)	90 (52.0)	71 (29.7)
Parous, mean (SD)	2.4 (1.2)	2.3 (0.98)	2.5 (1.3)
Birth state, n (%)			
Born in Utah	250 (60.7)	112 (64.7)	138 (57.7)
Not born in Utah	162 (39.3)	61 (35.3)	101 (42.3)
Racial groups, n (%)			
American Indian or Alaska Native	1 (0.24)	1 (0.58)	0
Asian	4 (0.97)	2 (1.2)	2 (0.84)
Native Hawaiian/Other Pacific Islander	2 (0.49)	0	2 (0.84)
Black or African American	3 (0.73)	1 (0.58)	2 (0.84)
White	363 (88.1)	151 (87.3)	212 (88.7)
Multiple races	23 (5.6)	12 (6.9)	11 (4.6)
Unknown	16 (3.9)	6 (3.5)	10 (4.2)
Hispanic ethnicity, n (%)	51 (12.4)	22 (12.7)	29 (12.1)
Smoking, n (%)	59 (14.3)	17 (9.8)	42 (17.6)
Endometriosis stage, n (%)			
Stage I	98 (23.8)	98 (56.6)	–
Stage II	26 (6.3)	26 (15.0)	–
Stage III	17 (4.1)	17 (9.8)	–
Stage IV	27 (6.6)	27 (15.6)	–
Endometriosis subtype, n (%)			
Superficial (SE)	143 (34.7)	143 (82.7)	–
Ovarian endometrioma (OE)	38 (9.2)	38 (22.0)	–
Deep Infiltrating (DE)	58 (14.1)	58 (33.5)	–

### Diagnostic accuracy measures

The percent agreement was relatively high for all comparison categories—87.6% for endometriosis diagnosis and between 83.7% and 91.0% for endometriosis subtypes. There was substantial agreement among overall endometriosis (*K* = 0.75) and SE subtype diagnoses (*K* = 0.65), moderate agreement in the OE subtype (*K* = 0.58), and slight agreement in the DE subtype (*K* = 0.17). All other measures for endometriosis diagnosis were above 0.80, indicating strong agreement. Accuracy measures in the SE and OE subtypes were relatively high. The DE subtype demonstrated the lowest accuracy measures, especially in sensitivity and Κ with moderate AUCR (Table 2).

**Table 2.** Diagnostic accuracy measures for endometriosis-associated diagnosis codes in the electronic health record.

Diagnostic accuracy measure	Endometriosis diagnosis	SE	OE	DE
Percent agreement	88% (84%, 91%)	84% (80%, 87%)	91% (88%, 94%)	87% (85%, 88%)
AUCR	0.88 (0.85, 0.91)	0.84 (0.81, 0.88)	0.87 (0.80, 0.93)	0.56 (0.52, 0.60)
Sensitivity	0.88 (0.83, 0.93)	0.86 (0.80, 0.92)	0.82 (0.68, 0.92)	0.12 (0.05, 0.21)
Specificity	0.87 (0.82, 0.91)	0.83 (0.78, 0.87)	0.92 (0.89, 0.94)	0.99 (0.98, 1.00)
NPV	0.91 (0.88, 0.94)	0.92 (0.89, 0.95)	0.98 (0.97, 0.99)	0.87 (0.86, 0.89)
PPV	0.83 (0.78, 0.88)	0.72 (0.67, 0.78)	0.51 (0.41, 0.61)	0.71 (0.38, 1.00)
Kappa (Κ)	0.75 (0.68, 0.81)	0.65 (0.59, 0.73)	0.58 (0.46, 0.68)	0.17 (0.05, 0.30)

SE, superficial endometriosis; OE, ovarian endometriomas; DE, deep infiltrating; AUCR, area under the receiver operating characteristic curve; NPV, negative predictive value; PPV, positive predictive value; Κ, Cohen’s kappa.

### Diversity analysis

We found no differences in diagnostic accuracy between specific populations, including Women of Color and White women as well as women of Hispanic ethnicity and non-Hispanic ethnicity (Table 3).

**Table 3.** Comparison of diagnostic accuracy measures of endometriosis diagnosis between populations.

Population	Percent agreement (95% CI)	AUCR (95% CI)	Kappa (*K*) (95% CI)
Women of color (n = 49)	90%(80%, 98%)	0.90(0.80, 0.98)	0.79(0.59, 0.96)
White (n = 363)	87%(84%, 91%)	0.87(0.84, 0.91)	0.74(0.67, 0.81)
Hispanic ethnicity (n = 51)	86%(76%, 94%)	0.87(0.77, 0.95)	0.72(0.53, 0.88)
Non-Hispanic ethnicity (n = 339)	88%(85%, 91%)	0.88(0.85, 0.92)	0.76(0.69, 0.82)

AUCR, area under the receiver operating characteristic curve; *Κ*, Cohen’s kappa.

### Error analysis

**Figure 1.**
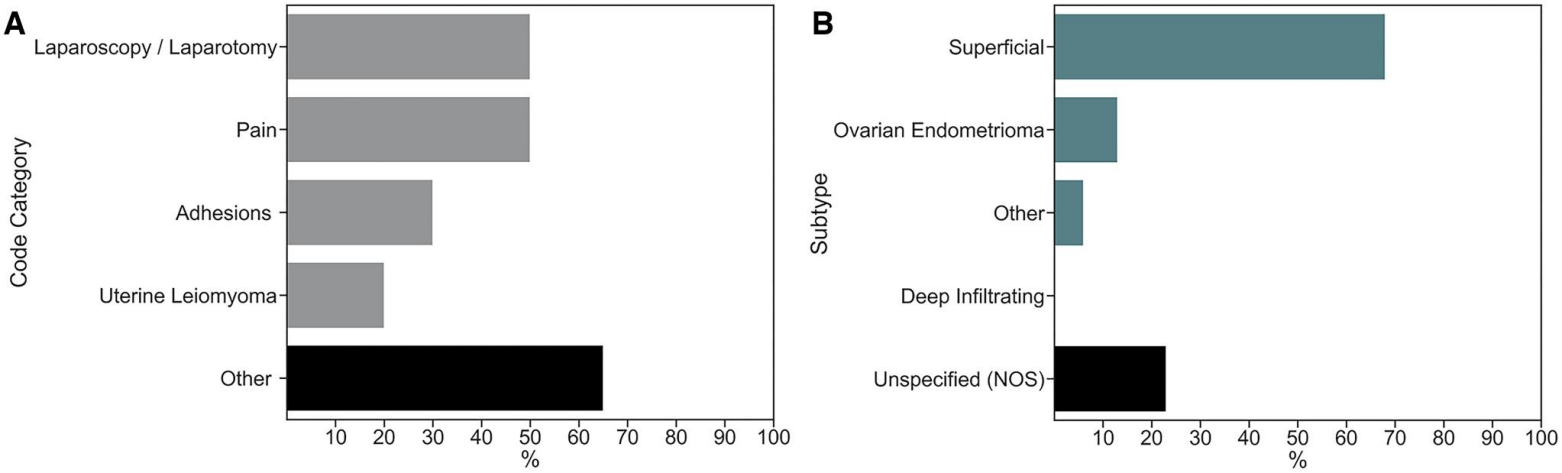
**Error analysis results.** (**A**) Codes present in the medical records of false negative patients (surgically confirmed disease not reported in the administrative health data at the time of the study). (**B**) Endometriosis subtypes diagnosed in the medical record of false positive patients (without disease per surgical confirmation but a record of disease in the administrative health data at the time of the study). NOS, not otherwise specified.

For the 20 false negative patients, Fig. 1A illustrates the diagnosis codes present in the administrative health data at the time of the ENDO Study. Codes indicating either a laparoscopy or laparotomy or pain were the most common in the false negatives, next to codes representative of other symptoms. For the 31 false positive patients, Fig. 1B illustrates the subtypes of endometriosis present in the administrative health data. SE was the most common subtype present in the false positives.

## Discussion

In this study, we found substantial agreement when comparing surgically confirmed endometriosis diagnoses with those found in administrative health data. The sensitivity and specificity of overall endometriosis diagnosis were relatively high. We additionally assessed endometriosis subtypes. While the sensitivity, specificity, and agreement for SE and OE were high, DE had lower sensitivity and agreement with high specificity. Our findings indicate clinically diagnosed endometriosis from administrative health data accurately reflects surgically confirmed diagnoses.

In the past, endometriosis research has suffered from small sample sizes—often attributable to lack of availability of participants with endometriosis diagnoses within a specific clinical setting (Eskenazi *et al.*, 2001; Shafrir *et al.*, 2021). To address this, some researchers have turned to medical records, or administrative health data, to identify women with endometriosis in greater numbers (Eskenazi *et al.*, 2001; Chao *et al.*, 2022; Penrod *et al.*, 2023). This is a viable method to increase the statistical power of a study; however, it has not been validated for endometriosis diagnosis. Previous studies have validated diagnoses found in the medical record for several conditions, including placenta accreta spectrum disorders, pulmonary hypertension, kidney disease, and heart failure (Quach *et al.*, 2010; Vlasschaert *et al.*, 2011; Wijeratne *et al.*, 2022; Jotwani *et al.*, 2024). This is the first study, to our knowledge, comparing endometriosis diagnoses from administrative health data to those confirmed surgically in a study setting. Our results demonstrated using diagnoses of endometriosis from administrative health data is both a viable and valid way to increase study size when researching endometriosis.

Previous studies have investigated the validity of self-reported endometriosis, as questionnaire-based research may be another method to increase statistical power in endometriosis research (Saha *et al.*, 2017; Shafrir *et al.*, 2021). Self-report relies on the memory of the participant to accurately report if they were diagnosed with endometriosis. Shafrir *et al.* (2021) performed a validation study of self-reported endometriosis diagnosis, comparing self-reported endometriosis with diagnoses from the medical record. They found women were able to recall their endometriosis diagnosis well, with 84% confirmation from self-report compared to the medical record. Saha *et al.* (2017) performed a different but similar study, comparing self-reported endometriosis with a national register. They found a high specificity of 97% but only moderate sensitivity of 62% of self-reported endometriosis. While these results indicate self-report can be a valid method of collecting endometriosis diagnosis status, these studies compared self-report to medical records. Our study went a step further, validating administrative health data, or medical records, against surgical diagnosis.

We found differing levels of sensitivity, specificity, and agreement among endometriosis subtypes. SE and OE were well documented in the medical records, resulting in relatively high agreement, sensitivity, and specificity. The sensitivity for DE was low, meaning there were many cases of DE subtype not documented in the medical records with diagnosis codes. However, the specificity of DE was very high, meaning when a case of DE was recorded, this was almost always correct. This lack of documentation of DE has been reported previously (Shafrir *et al.*, 2021). Vanhie *et al.* (2016) proposed standardized surgical reporting forms for DE to address deficient standardization and incomplete reporting. From an epidemiological standpoint, high specificity is ideal as we want to be sure that cases studied are true cases. In the future, greater documentation of endometriosis subtypes, especially DE, in administrative health data could allow for more in-depth analyses.

While our study has many strengths, we also acknowledge the limitations of this study. We did not include clinical notes in this study as we were only looking at structured administrative health data. Clinical notes have the potential to hold more detailed information, including endometriosis subtype. However, these are traditionally more difficult to utilize for research, often requiring natural language processing pipelines. Additionally, given that participants whose administrative health records we utilized were also part of the ENDO Study, the surgeons may have been more vigilant in diagnostic coding due to the operative forms they completed for the ENDO Study, which may have led to increased validity. However, the surgeries and administrative health data reporting was independent of the ENDO Study with all surgeons (n = 152) within the geographic catchment area eligible to perform ENDO Study surgeries and instructed not to change their practice in any way, and thus we believe our study captures what happens in a usual pelvic laparoscopy in this system. This study may have limited generalizability to the USA overall, as most of the study population was white (88.1%). However, we did evaluate diagnostic accuracy among diverse populations, albeit with smaller sample sizes. Despite the limitations, this study was able to compare surgically confirmed diagnoses from a comprehensive research study with diagnoses from administrative health data as opposed to self-reported diagnoses. The limited exclusion criteria of the original ENDO Study provided a wider range of patients for our current study, including women with many different surgical indications—not just pelvic pain or infertility.

We observed substantial agreement between administrative health data and surgically confirmed endometriosis diagnoses overall, and for SE and OE subtypes. Our findings for nearly perfect specificity but low sensitivity for DE corroborates prior research and suggest that medical record-based DE diagnoses may be suitable for etiologic studies—cases identified are true cases—but not for surveillance or detection studies—not all true cases are identified. These findings may provide reassurance to researchers using administrative health data to assess risk factors and long-term health outcomes of endometriosis.

## Supplementary Material

deae281_Supplementary_Figure_S1

deae281_Supplementary_Table_S1

## Data Availability

The data underlying this article cannot be shared publicly due to protected health information and Health Insurance Portability and Accountability Act compliance. The data will be shared on reasonable request to the corresponding author. The reproducible code for this analysis can be accessed via GitHub at: https://github.com/amberkiser/Endometriosis-Dx-in-Administrative-Data.
