# Supplementary material for: Validation of administrative health data for the identification of endometriosis diagnosis
Source: Hum Reprod. 2024 Dec 20;40(2):289–95. doi: 10.1093/humrep/deae281 (PMC11788219; doi:10.1093/humrep/deae281)
Supplement: deae281_Supplementary_Figure_S1 [file deae281_supplementary_figure_s1.pdf]

Human Reproduction, 2024, 1–1
https://doi.org/10.1093/humrep/deae281
Supplementary Data

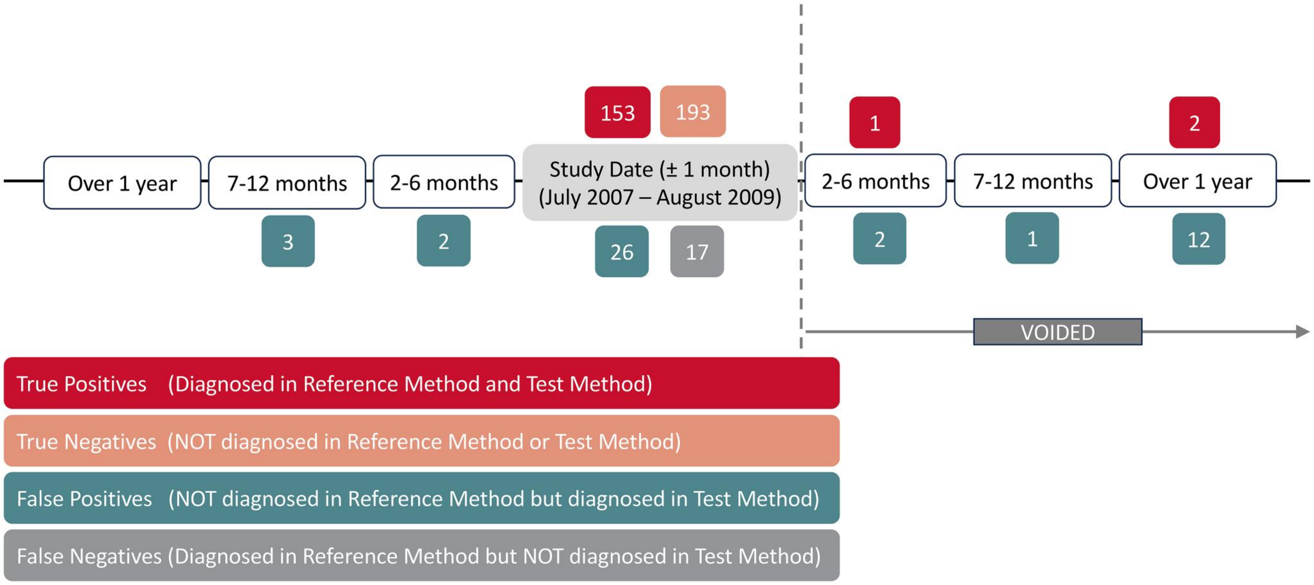

Supplementary Figure S1. Timeline of endometriosis diagnoses. Reference method refers to diagnoses from the Endometriosis, Natural history, Diagnosis, and Outcomes (ENDO) Study. Test method refers to diagnoses from administrative healthcare data.
