# Supplementary material for: Validation of administrative health data for the identification of endometriosis diagnosis
Source: Hum Reprod. 2024 Dec 20;40(2):289–95. doi: 10.1093/humrep/deae281 (PMC11788219; doi:10.1093/humrep/deae281)
Supplement: deae281_Supplementary_Table_S1 [file deae281_supplementary_table_s1.pdf]

OXFORD
human reproduction
Human Reproduction, 2024, 1–1
https://doi.org/10.1093/humrep/deae281
Supplementary Data

Supplementary Table S1. International Classification of Diseases (ICD) codes for endometriosis subtype diagnoses.

ICD 9	ICD 10	Description	Subtype
617	N80	Endometriosis	Superficial
617.0	N80.0	Endometriosis of uterus	Superficial
617.1	N80.1	Endometriosis of ovary	Ovarian endometrioma
617.2	N80.2	Endometriosis of fallopian tube	Superficial
617.3	N80.3	Endometriosis of pelvic peritoneum	Superficial
617.4	N80.4	Endometriosis of rectovaginal septum and vagina	Deep infiltrating
617.5	N80.5	Endometriosis of intestine	Deep infiltrating
617.6	N80.6	Endometriosis in scar of skin	Other
617.8	N80.8	Endometriosis of other sites unspecified	Other
617.9	N80.9	Endometriosis, site unspecified	Other

© The Author(s) 2024. Published by Oxford University Press on behalf of European Society of Human Reproduction and Embryology.
This is an Open Access article distributed under the terms of the Creative Commons Attribution License (https://creativecommons.org/licenses/by/4.0/
), which permits unrestricted reuse, distribution, and reproduction in any medium, provided the original work is properly cited.
Human Reproduction, 2024, 00, 1–7
https://doi.org/10.1093/humrep/deae281
Original Article
